# Observation on the effectiveness and safety of sodium bicarbonate Ringer’s solution in the early resuscitation of traumatic hemorrhagic shock: a clinical single-center prospective randomized controlled trial

**DOI:** 10.1186/s13063-022-06752-5

**Published:** 2022-09-30

**Authors:** Jizhe Zhang, Dong Han, Kun Zhang, Weiqiang Guan, Li Li, Zhengtao Gu

**Affiliations:** 1 Department of Treatment Center For Traumatic Injuries, The Third Affiliated Hospital of Southern Medical University, Guangzhou, Guangdong China; 2 Guangdong Provincial Key Laboratory of Bone and Joint Degenerative Diseases, Academy of Orthopaedics, Guangdong Province Guangzhou, China; 3 Orthopaedics Hospital of Guangdong Province, Guangzhou, Guangdong China; 4 Department of Quality Control and Evaluation, The Third Affiliated Hospital of Southern Medical University, Guangzhou, Guangdong China

**Keywords:** Traumatic hemorrhagic shock, Fluid resuscitation, Sodium bicarbonate Ringer’s solution, Lactate clearance

## Abstract

**Background:**

Traumatic hemorrhagic shock (THS) is the main cause of death in trauma patients with high mortality. Rapid control of the source of bleeding and early resuscitation are crucial to clinical treatment. Guidelines recommend isotonic crystal resuscitation when blood products are not immediately available. However, the selection of isotonic crystals has been controversial. Sodium bicarbonate Ringer solutions (BRS), containing sodium bicarbonate, electrolyte levels, and osmotic pressures closer to plasma, are ideal. Therefore, in this study, we will focus on the effects of BRS on the first 6 h of resuscitation, complications, and 7-day survival in patients with THS.

**Methods:**

/design.

This single-center, prospective, randomized controlled trial will focus on the efficacy and safety of BRS in early THS resuscitation. A total of 400 adults THS patients will be enrolled in this study. In addition to providing standard care, enrolled patients will be randomized in a 1:1 ratio to receive resuscitation with BRS (test group) or sodium lactate Ringer’s solution (control group) until successful resuscitation from THS. Lactate clearance at different time points (0.5, 1, 1.5, 3, and 6 h) and shock duration after drug administration will be compared between the two groups as primary end points. Secondary end points will compare coagulation function, temperature, acidosis, inflammatory mediator levels, recurrence of shock, complications, medication use, and 7-day mortality between the two groups. Patients will be followed up until discharge or 7 days after discharge.

**Discussion:**

At present, there are still great differences in the selection of resuscitation fluids, and there is a lack of systematic and detailed studies to compare and observe the effects of various resuscitation fluids on the effectiveness and safety of early resuscitation in THS patients. This trial will provide important clinical data for resuscitation fluid selection and exploration of safe dose of BRS in THS patients.

Trial registration.

Chinese Clinical Trial Registry (ChiCTR), ChiCTR2100045044. Registered on 4 April 2021.

**Supplementary Information:**

The online version contains supplementary material available at 10.1186/s13063-022-06752-5.

## Introduction

### Background

Severe trauma is a major global public health problem, causing an estimated more than 5.8 million deaths worldwide each year, accounting for one in ten of all deaths [1]. Blood loss due to trauma is the most important cause of death and the leading cause of death among people under 40 years of age worldwide and can cause significant economic losses. Traumatic hemorrhagic shock (THS) refers to the pathophysiological process in which trauma leads to massive loss of effective circulating blood in patients, resulting in tissue hypoperfusion, cell metabolism disorder, and organ function damage [2]. In the early stages of trauma, control of bleeding prevents death, fluid resuscitation improves tissue perfusion, and prevention of traumatic coagulopathy (TIC) reduces secondary organ failure and ultimately minimizes mortality [3–10]. Based on the above, we found that the key to clinical treatment of THS patients is rapid control of the source of bleeding, early fluid resuscitation, and restoration of intravascular volume and oxygen carrying capacity [11]. However, the choice of optimal resuscitation fluid for THS patients is still a controversial issue. Finding fluid suitable for early resuscitation is a key problem that needs to be solved urgently in clinical treatment of THS patients.

Over the past decades, many clinical studies have confirmed that early fluid resuscitation using blood products is the best option. However, blood products are often difficult to obtain immediately in the early stages of trauma. Studies have shown that pre-hospital plasma use is not associated with survival benefits in rapid ground support [12, 13]. Hypertonic crystalloids, hypotonic crystalloids, and artificial colloids have significant component differences from plasma. Their use follows many risks, such as exacerbating acidosis, causing impairment of renal function, coagulation function, platelet function, and so on [14–29]. Therefore, they are not the most optimal option. Guidelines recommend using isotonic crystalloid resuscitation in cases where blood products are not immediately obtainable [1, 25, 30]. Studies show that isotonic crystalloids with similar components to plasma become the primary option for patients with THS, except for traumatic brain injury (TBI), and fluids with the potential to restore pH may be beneficial for resuscitation [24, 25, 27, 28, 31].

Currently, the most commonly used isotonic crystalloids in clinical treatment are sodium lactate Ringer’s solution (LRS) and sodium acetate Ringer’s solution (ARS), which are distinguished by their fluid components. Lactate and acetate are metabolized in the liver and kidneys to form CO_2_ and H_2_O to maintain acid–base balance and self-clearance. THS patients lose too much blood, resulting in reduced blood perfusion to the liver and kidneys. Additional input of lactate and acetate not only increases the burden on the liver and kidneys but also aggravates the degree of acidosis due to its accumulation, accelerating the process of “bloody vicious circle” making the patient’s prognosis worse. Therefore, use of LRS and ARS is not conducive to patient resuscitation and will affect the efficacy of treatment. Sodium bicarbonate Ringer’s solution (BRS) contains the bicarbonate buffering system and its pH, electrolyte levels, and osmolality are similar to plasma. Studies show that BRS can directly participate in acid–base metabolism, modify base excess (BE) values, maintain acid–base balance, decrease lactate levels, and diminish lung cell apoptosis [32–38]. In contrast, we found that BRS, which is more similar to the plasma component, is expected to be the optimal option. Additional file 1 shows the differences between these fluid components.

At present, there are no large-scale clinical trials of early fluid resuscitation in THS patients using BRS, and systematic and detailed comparisons of the efficacy and safety of these isotonic crystals are lacking. We therefore designed this single-center, prospective, randomized controlled trial.

### Objectives

By comparing the differences in duration of shock and the lactic acid clearance rate at each time point when early fluid resuscitation was performed in the test group and control group. To identify whether early fluid resuscitation using BRS can reduce the complication rate and improve 7-day survival in patients with THS.

## Methods

### Study design

This study is a clinical single-center, prospective, superiority, parallel-group, randomized controlled trial in patients with THS. The study will be conducted at the Department of Treatment Center for Traumatic Injuries and Emergency Intensive Care Unit (EICU) of the Third Affiliated Hospital of Southern Medical University, China. Patients who meet the eligibility criteria and obtain informed consent will be assigned to the test group or control group in a 1:1 ratio based on the size of the random number allocated to them. Patients will be resuscitated with the drug according to the study arm, and only this drug will be used for resuscitation in the early fluid resuscitation (6 h) period. Approval has been obtained from the Institutional Ethics Committee (IEC) of the Chinese Ethics Committee of Registering Clinical Trials. This study complies with the Declaration of Helsinki and the relevant regulations for clinical research in China. This trial has been registered as ChiCTR2100045044.

**Fig. 1:**
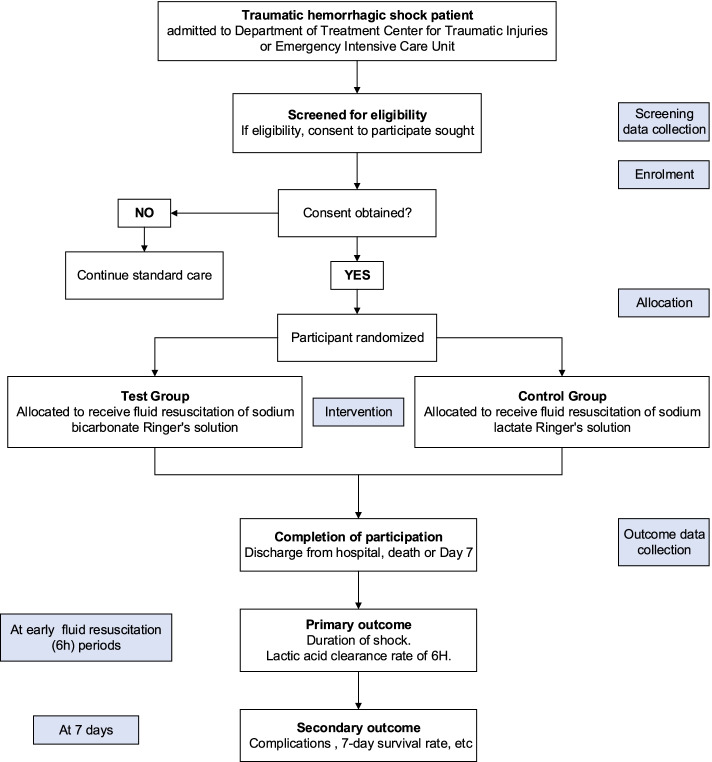
Study scheme

**Fig. 2:**
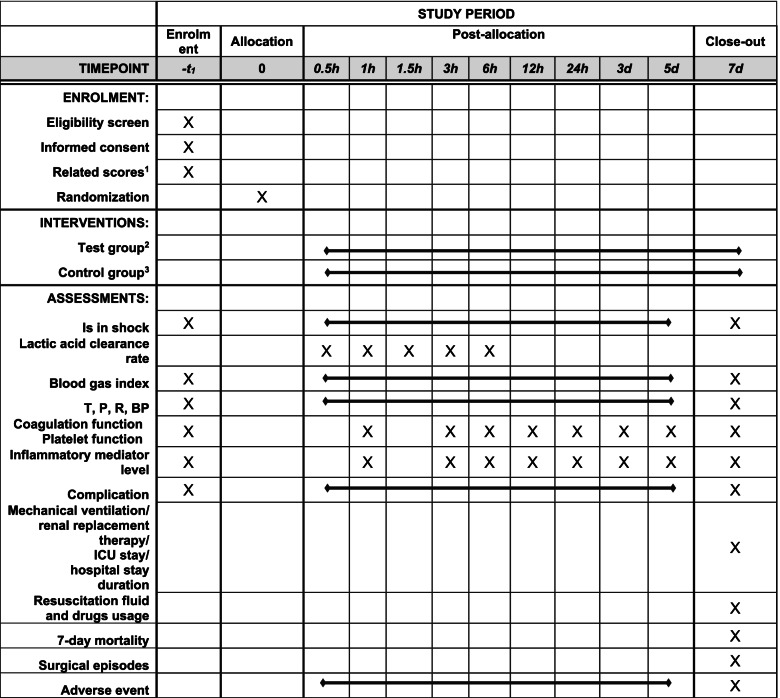
Schedule of enrolment, interventions, and assessments. ^1^Shock Index, APACHE II, GCS score, ISS score, AIS score, NISS score, SOFA score, CRAMS score. ^2^Sodium bicarbonate Ringer’s solution group. ^3^Sodium lactate Ringer’s solution group

The protocol is written in accordance with the Standard Protocol Items: Recommendations for Interventional Trials (SPIRIT) guidelines, shown in Additional file 2. Figure 1 (study scheme) shows an overview of the study process. Figure 2 shows the schedule of enrolment, interventions, and assessments.

### Eligibility criteria

#### Inclusion criteria

1. Patients with THS [39, 40] (Table 1 shows the diagnostic criteria for THS)
2. Age ≥ 18 years

**Table 1:** The diagnostic criteria for THS. Patients who at the same time meet Clinical history, Vital signs^1^, and any three of Vital signs^2^ will be diagnosed as THS

Clinical history	Vital signs^1^	Vital signs^2^
There are traumas that cause hemorrhage (such as road traffic injuries)	(1) Systolic blood pressure (SBP) < 80 mmHg, or mean arterial pressure < 65 mmHg, or a decrease of ≥ 40 mmHg from baseline. (2) Lactate level > 2 mmol/L.	(1) Consciousness changes, includes unresponsiveness, disorientation, and confusion. (2) The skin is wet and cold with vasoconstriction and cyanosis floridis, capillary filling time > 2 s. (3) Urine output < 0.5 ml/kg/h. (4) Pulse rate > 100 beats/min or untouchable, shock index > 1.0.

### Exclusion criteria

1. Traumatic brain injury (TBI) and brain stem failure
2. Severe cardiopulmonary dysfunction
3. Severe hepatorenal insufficiency
4. Hypermagnesemia
5. Hypothyroidism
6. Used acid correction drugs
7. Minorities: pregnant, lactating, and prisoners
8. Treating physician believes the patient is not suitable for the trial
9. Inability to obtain consent
10.   Patients automatically gave up treatment or died during the study

#### Stopping criteria

1. Receive blood transfusion, intervention, or surgery within 1 h after admission
2. Patients who died within 1 h after intervention
3. Patients with serious adverse events or unexpected events in the clinical process
4. Patients who did not complete the trial protocol observation period regardless of when or why they quit
5. Patients ask to be withdrawn from the study
6. Investigator termination of the study for any reason or any pathological event, adverse event, or change in the subject’s medical condition that leads the physician to conclude that continued participation in the study is not in the best interest of the patient

#### Termination study conditions

1. If a serious adverse event (SAE) occurs in the trial, the trial should be terminated in time
2. If a major error in the clinical trial scheme is found in the trial, or there is a serious deviation in the scheme’s implementation. It is difficult to evaluate the efficacy of the drug. The trial should be suspended
3. In the trial, it was found that the drug treatment effect was not effective (there was no therapeutic effect compared with the control group, the difference of each observation index was not statistically significant), it did not have clinical value, and the trial should be suspended
4. The sponsor is asked to stop the trial

#### Blinding

This trial is an open-blinding trial, because patients are usually unconscious and the packaging of the two trial drugs (BRS and LRS) differs significantly.

### Study procedures

#### Screening procedures

Investigators will use eligibility criteria to identify eligible adults with THS as soon as possible after the patient arrives at the hospital. If the patient meets the criteria for enrollment, the investigator will ask the patient’s legal representative for informed consent to participate in the trial. The screening diary is completed every 2 weeks, and the record investigator will record all patients deemed eligible for the trial. The log will include age, gender, inclusion/exclusion criteria, and other reasons for failure to enter the study arm. The screening log data will be reviewed regularly.

#### Randomization procedure

Enrolled patients will be assigned to the study arm in a 1:1 ratio based on the size of the assigned numbers via a computer-generated random number table within 10 min of admission. Before the study began, a random envelope was prepared for each bed. The envelope contains a card printed with a number (from the random number table). Once the patient is confirmed eligible to participate in the study and informed consent is obtained, the envelope will be opened and unblinding by the investigator immediately after the patient arrives at the bedside. Patient study arm is determined based on the size of the numbers on the card to ensure there is no delay in treating the patient. The investigator will not know the study arm until unblinding takes place and each envelope will be used only once.

#### Schedule of intervention

All patients will be managed according to the standard diagnosis and treatment process, regardless of whether they participate in the trial or not. After randomization, enrolled patients will receive intervention at the set time point, as showed in Fig. 2, and they will be followed up for 7 days.

#### Standard care

All patients are treated in accordance with the treatment of THS: Priority is relieving the life-threatening situation so that the injury can be preliminarily controlled, and then the follow-up treatment is carried out. The “saving life first, the protective function second, first emphasizing and then light, first urgent and then slow” [41] was followed. Damage-controlled resuscitation (DCR) strategy was adopted: immediately establish two or more venous channels, indwell the arterial sheath, perform ECG monitoring, and monitor the patient’s arterial blood pressure at the same time, implement permissible hypotension before major bleeding is controlled, and maintain SBP at 80–90 mmHg. Restrict the input of a large amount of liquid. Standard care includes:

(A) Rapid assessment of injury
(B) Continuous monitoring of vital signs
(C) Control of bleeding
(D) Respiratory support
(E) Improvement and continuous follow-up of relevant laboratory and imaging examinations
(F) Control of body temperature
(G) Pain relief
(H) Prevention and treatment of traumatic coagulopathy
(I) Prevention and management of acidosis
(J) The use of vasoactive drugs and positive inotropic drugs when necessary
(K)  Damage control surgery
(L) Other symptomatic supportive treatment

#### Initiation of study care

When patients are enrolled in the trial, they will receive standard care and be resuscitated with fluids depending on the study arm. The first 6 h of resuscitation will be administered with BRS or LRS for early resuscitation only. After early resuscitation, the blood products will be given for resuscitation depending on the patient’s condition. Moreover, the patient’s blood sample will be collected before the fluid resuscitation. After the patient enters the study arm, only the fluid type corresponding to the study arm is used for resuscitation. Given that fluid is 500 ml/time, the administration speed is ≤ 10 ml/kg/h, and the total amount is less than 3L during the resuscitation for the first 6 h. Albumin can be used for resuscitation when necessary.

**Fig. 3:**
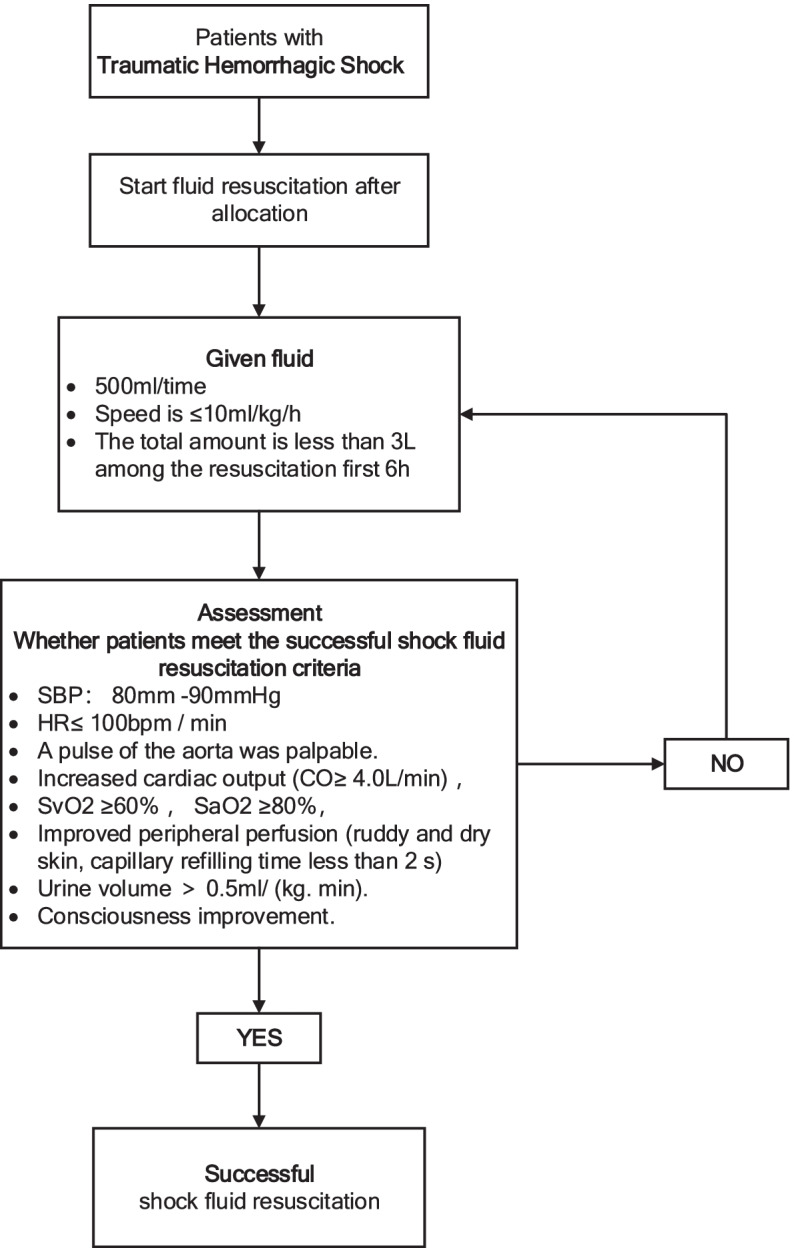
Drug application process

Enrolled patients will be block randomized to either study arm (Fig. 3 shows the drug application process).

Clinical criteria of successful shock resuscitation include:

(A) SBP was maintaining at 80–90 mmHg
(B) Heart rate was less than or equal to 100 bpm/min
(C) A pulse of the aorta was palpable
(D) Increased cardiac output (greater than or equal to 4.0L/min)
(E) Improved peripheral perfusion (ruddy and dry skin, capillary refilling time less than 2 s)
(F) Mixed venous oxygen saturation is greater than or equal to 60% and arterial oxygen saturation greater than or equal to 80%
(G) The urine volume was greater than that of 0.5 ml/(kg min)
(H) Consciousness improvement
(I) Lactate level ≤ 2 mmol/L

##### Test group

For standard care, damage-controlled resuscitation strategy is used, only BRS during fluid resuscitation is used, 500 ml/time is used, dosing rate of ≤ 10 ml/kg/h is used, SBP at 80–90 mmHg is maintained, and the total amount is less than 3L during the resuscitation for the first 6 h. Investigators will evaluate whether the patient’s shock state is corrected before each administration. If the resuscitation is successful, the drug will be stopped, and if the shock persists, the drug will be continued.

##### Control group

For standard care, damage-controlled resuscitation strategy is used, only LRS during fluid resuscitation is used, 500 ml/time is used, dosing rate of ≤ 10 ml/kg/h is used, SBP at 80–90 mmHg is maintained, and the total amount is less than 3L during the resuscitation for the first 6 h. Investigators will evaluate whether the patient’s shock state is corrected before each administration. If the resuscitation is successful, the drug will be stopped, and if the shock persists, the drug will be continued.

#### Cessation of study care

For this study, successful shock resuscitation (end of study care) will be defined as the last intravenous injection of BRS /LRS investigator who evaluated the patient’s shock state and believed that shock resuscitation had achieved success.

#### Provisions for post-trial care

This study has insured the subjects who participated in the trial.

### Outcomes

#### Primary endpoint

(A) Duration of shock
(B) Lactic acid clearance rate of 6 h

### Secondary endpoints

(A) The amount of drug use, the total amount of resuscitation fluid, and the use of vasoactive drugs was compared in each study arm.
(B) The total number of deaths and mortality was compared. The Kaplan–Meier survival curve was drawn, and the 7-day mortality of patients in each study arm was compared.
(C) The levels of inflammatory mediators (PLA2, TNF- α, IL-6, PCT, CRP) were compared in each study arm.
(D) The recurrence rate of shock and the complications of survival cases (ARDS, AKI, TIC, MODS, etc.) were compared among the study arms.
(E) Blood coagulation and platelet function (PT, APTT, TT, INR, ACT, Fib, VEM) were compared among different study arms and before and after treatment.
(F) Body temperature and acidosis (T, PH, BE, Lac, PaCO_2_, HCO_3_^−^) was compared among different study arms and before and after treatment.
(G) The mechanical ventilation time, EICU hospitalization time, and total hospitalization time of patients in each study arm was compared.

### Detailed outcome measures collected

#### Duration of shock

The time point at which the patient began in the shock state and the time point at which shock resuscitation was successful during the entire trial process will be recorded, and the shock duration will be calculated.

#### Lactic acid clearance rate of 6 h

The lactic acid in the blood gas results is recorded before fluid resuscitation (0 h) and after resuscitation (0.5, 1, 1.5, 3, and 6 h). The lactic acid clearance rate at the above is calculated based on the time point change in the lactic acid.

#### Temperature and acidosis

The patient’s body temperature and blood samples will be collected for blood gas before resuscitation (0 h) and after resuscitation (0.5 h, 1 h, 1.5 h, 3 h, 6 h, 12 h, 24 h, 3 days, 5 days, and 7 days). The acidosis at the above time point in the blood gas results is recorded.

#### Coagulation function, platelet function, and inflammatory mediator level

Blood samples will be collected before resuscitation (0 h) and after resuscitation (1 h, 3 h, 6 h, 12 h, 24 h, 3 days, 5 days, and 7 days) for analysis and recording of coagulation function, viscoelastic method (VEM), and inflammatory mediator level.

#### Shock recurrence rate

The time point of shock recurrence and the cause of shock recurrence will be recorded throughout the study process, and the shock recurrence rate will be calculated.

#### Complications

Complications (ARDS, AKI, TIC, MODS, etc.) that occurred during the entire study process will be recorded and confirmed through corresponding diagnostic methods.

#### Duration of mechanical ventilation, renal replacement therapy

The days of mechanical ventilation, hemodialysis, or hemofiltration are calculated and recorded.

#### Resuscitation fluid and drug usage

The total volume of different fluids used before admission and after entering the study until the 7th day is recorded, including crystals, colloids, blood products, hemostatic drugs, and vasoactive drugs.

#### EICU days and length of stay

Hospital stays and length of EICU days will be recorded. If the patient is hospitalized at any time of the day, that day will be regarded as the hospitalization day. If the patient is in the EICU at any point on the calendar day, that day will be considered EICU day.

#### Surgical episodes

The name, start time, duration, and cause of all surgical episodes will be recorded. In addition to major surgical procedures, this also includes interventional radiology and bedside surgery and operations.

#### Data and safety monitoring

Investigators need to fill in the data collected in the case report form (CRF) according to the requirements of the research program. At the end of the study, the investigator will submit the case reports of all patients selected for this study to the data management center, which should be complete and signed. The consistency of the collected case reports table data will be checked. The inconsistent data will be questioned, and the responsible doctor will be required to clarify. Before starting the trial, unified training will be given to the investigator to clarify the purpose of the study and ensure that the same test methods are used in the trial. The investigator must research in strict accordance with the requirements of the research program. Patients are randomly and distributed by statisticians and data management centers, and the doctor is responsible for in putting information into the case report form. The CRF supervisor will check the completeness and accuracy of the CRF and guide the research center staff to make necessary modifications or supplements. The CRF is sent to the data processing by the regional supervisor, one copy is retained in the research center, and the other one is used as an attachment to the supervisor’s work. CRF will hand it over to a reliable medical data processor for data entry. Data items in CRF will be entered into the research database using a multiple-input method with secondary input audits. Text items (such as comments) can only be checked manually after entering once from CRF. The data manager then systematically checks the information entered into the database using error messages printed from the confirmer program and the database list. The database will be locked after it is declared complete and correct. After that, any changes to the database can only be achieved with the written consent of clinical research leaders, research statisticians, and data managers.

#### Plans to give access to the full protocol, participant-level data, and statistical code

Full protocol, anonymous participant-level data, and statistical code will be shared on reasonable request by scientists.

#### Sample size

The trial design of this study was a positive drug, parallel-group, and superiority verification. The primary endpoint is the duration of shock and lactic acid clearance rate during the early fluid resuscitation period (6 h). Both primary endpoints were required to be met simultaneously, and Bonferroni correction was used as appropriate. The alpha value = 0.025 (one-sided), power = 0.80 were set. The total sample size was finally determined to be 400 cases with 200 cases in each study arm.

The calculations were as follows:

- Lactic acid clearance rate: According to a study by Wang et al., the mean value of lactic acid clearance rate after 6 h of fluid resuscitation in the control group was 32% with a standard deviation of 10%, and the expected lactic acid clearance rate after 6 h of fluid resuscitation in the test group was 35% with an equal standard deviation for both study arms [42]. The calculated sample size was 176 cases for each study arm, and a total of 392 cases were required based on 10% of the loss to follow-up.
- Duration of shock: According to the pre-experimental study, the duration of shock in the control group was 3 h, and in the test group the duration of shock was 1.5 h, with a standard deviation of 4 h. Assuming equal standard deviation for both study arms, the sample size calculated for both study arms was 113 cases, and a total of 252 cases were required based on 10% of the loss to follow-up.

#### Statistical analysis

This trial was performed using SPSS 26.0 for data analysis. A general linear model was used to correct for selection bias between the two study arms. Categorical data were described as percentages. The measurement data were tested for normality, approximately normal or normally distributed measures were expressed as mean ± standard deviation (x ® ± s), and non-normally distributed measures were expressed as median (quartiles). The chi-square test/ Fisher’s precision probability test was used for categorical data. Paired *t*-test/rank sum test/one-way repeated measures ANOVA/two-way repeated measures ANOVA was used for the measurement data. Kaplan–Meier survival curves were plotted for survival analysis of patients, and differences between the two study arms were compared using the log rank test. Multiple regression analyses were selected to analyze the interrelationships between the factors. The missing data were handled using the last observation carry forward (LOCF) method, and sensitivity analysis was performed using the multiple imputation method. *P* < 0.05 was statistically significant.

#### Interim analyses

No interim analysis will be performed.

#### Pharmacovigilance plan

An adverse event (AE) is defined as any adverse medical event that occurs to a patient, which does not necessarily have a causal relationship with the treatment. Therefore, an adverse event can be any discomfort or unconscious signs (including abnormal laboratory results), symptoms, or disease after using the drug, whether or not it is considered drug-related. Such incidents can occur when used by the drug instructions or due to accidental or intentional overuse, drug abuse, or drug withdrawal. Any pre-existing condition or deterioration of the disease is also considered to be adverse. If it meets any of the following criteria, it will be considered a serious adverse event (SAE): the subjects died, endanger one’s life, extended hospital stay / ICU time, persistent or apparent disability/loss of function, an important medical event that requires medication or surgery to avoid serious consequences. In the event of a serious adverse event, whether or not the incident is related to the research drug or reference substance (if applicable), the doctor should notify the Research Supervision team within 24 h of the incident. The specimen management system for patients with adverse events will actively treat the patients and terminate the trial in the event of adverse events, and the remaining drugs will be uniformly recovered and centrally destroyed. The patient’s blood, body fluids, secretions, and other test-related specimens will be destroyed and recorded under the supervision of the main person in charge and the inspectorate.

#### Data management

Investigator will ensure that the privacy of clinical trial subjects is maintained. In all the documents of the sponsor, the identity of the clinical trial subject can only be determined by trial patient number and initials, but the full name of the subject cannot be indicated. The investigator will properly keep the names and addresses of the subjects in the clinical trial and the corresponding enrollment forms. These enrollment forms will be kept strictly confidential by the investigator. In addition, the investigator will properly keep the samples taken by the subjects during the study (including tissues, blood samples), and subject samples are strictly limited to research use. The investigator shall keep the information of this study strictly confidential and at the same time require other trial participants and the ethics committee to adopt the same confidentiality measures and shall not disclose it to any third party or use it for personal use without the written permission of the sponsor. The principal investigator shall strengthen security and information confidentiality management for those exposed to drugs, technical information, and/or test data and results and shall not disclose any information or information for personal use. The data of drug clinical trials include all data of clinical trials, and the project manages clinical trial data. The data includes two forms: written data and electronic data. Special computer stores the electronic version data, and the power-on password is set. The dedicated computer is operated and managed by the data administrator. The written data is kept by the counter and locked. The key is kept by the data manager and the person in charge of the research room.

#### Plans for communicating important protocol amendments to relevant parties (e.g., trial participants, ethical committees)

If important amendments to the trial protocol are required, regulatory approval is needed before implementation.

#### Dissemination plans

After the statistical analysis of this trial is completed, the publication is planned in several international publications. All data and protocols will be published in the Clinical Trial Management Public Platform (ResMan) after the results are published.

## Discussion

THS is a common serious complication of severe trauma. Without aggressive and effective treatment, it can lead to a series of serious consequences (e.g., uncorrected acidosis, hypothermia, TIC) and extremely high mortality, requiring continuous and comprehensive treatment. To improve the success rate of THS surgical rescue, shorten the time of shock, improve the prognosis, and reduce the economic burden of patients are urgent clinical problems to be solved. What kind of fluid to choose in early resuscitation and what kind of resuscitation method to use can reduce mortality and complications are the hot spots of current attention. Moreover, there is a lack of systematic and detailed studies on the effect of different infusion on the efficacy and safety of resuscitation in THS patients, and there is no unified standard to guide clinical treatment.

Since the composition of BRS is more similar to plasma, BRS has shown its advantages over other liquids in the current study. BRS is shown to improve metabolic acidosis, maintain acid–base balance, prevent arrhythmias, and supplement circulating blood volume in the perioperative period [36–38]. G Tom Shires’ and Laura Boomer’s studies have shown that using BRS could significantly reduce apoptosis levels in lung and liver tissue and inhibit pro-inflammatory responses [32, 33]. Shinji Oikawa’s study showed that BRS has the positive capability to maintain PLT function [34]. A small clinical trial with a sample size of 50 showed that resuscitation with BRS improved coagulation function, lactate levels, pH, and hemorheological indices in patients [35].

However, these trials are mainly in vitro or perioperative studies, and there is a lack of large-scale credible clinical studies to demonstrate the use of BRS in early fluid resuscitation in THS. The only study in THS patients was small in size, lacked continuous observation of the entire resuscitation process, lacked subgroup analysis of different injury types, and had simple observation parameters. Therefore, a large clinical trial with sufficient sample size is needed, not only to continuously observe the entire resuscitation process and prognosis, but also to perform subgroup analysis of patients with different injuries, and to have more comprehensive observation parameters to confirm the benefits and limitations of BRS in the early resuscitation of THS patients.

At present, more studies are related to the endpoint of resuscitation in septic shock, but THS resuscitation is still lacking. In the treatment of sepsis, a 6-h bundle treatment and a 6-h resuscitation goal have been proposed to minimize mortality in patients with sepsis. It has been shown that lactate clearance rate is also associated with prognosis and lower mortality in shock patients, with higher lactate clearance rate at 6 h being associated with better prognosis. In addition, the study showed that the return of BE to normal within 6 h was positively associated with a good patient outcome. Therefore, different time points within 6 h (0.5, 1, 1.5, 3, and 6 h) were selected as the key time points of the experiment. This has not been studied in fluid resuscitation in THS patients and is an innovation of this trial.

Based on this, we designed this trial to explore the key role of resuscitation fluid selection in early resuscitation, explore the mechanism of different resuscitation fluids from multiple aspects, clarify the effectiveness of the new resuscitation fluid BRS in early resuscitation of THS, and explore the safe dosage and time point of BRS. It will provide a new reference and idea for clinical treatment in the future.

### Trial status

The current protocol version is 5.0 (date September 6, 2022). Recruitment began on 1 May 2021 and is expected to end in June 2024.

## Supplementary Information

**Additional file 1.**

**Additional file 2.**

## Data Availability

Data collection for this study will be accomplished using a paper CRF to capture data prospectively and transferred to an electronic data capture system. The dataset will be open to access and share on the Clinical Trial Management Public Platform.

## Abbreviations

AKI: Acute renal failure
ARDS: Acute respiratory distress syndrome
ACT: Activated clotting time
AE: Adverse event
BE: Base excess
CRP: C-reactive protein
CRF: Case report form
EICU: Emergency intensive care unit
THS: Traumatic hemorrhagic shock
TIC: Traumatic coagulation
TBI: Traumatic brain injury
IL-6: Interleukin-6
IEC: Institutional Ethics Committee
MODS: Multiple organ dysfunction syndromes
PCT: Procalcitonin
PLA2: PhospholipaseA2
SBP: Systolic pressure
SAE: Serious adverse event
TNF- α: Tumor necrosis factor- α
VEM: Viscoelastic method
