# Supplementary material for: Observation on the effectiveness and safety of sodium bicarbonate Ringer’s solution in the early resuscitation of traumatic hemorrhagic shock: a clinical single-center prospective randomized controlled trial
Source: Trials. 2022 Sep 30;23:825. doi: 10.1186/s13063-022-06752-5 (PMC9523956; doi:10.1186/s13063-022-06752-5)
Supplement: Supplementary file 1 — Additional file 1. [file 13063_2022_6752_MOESM1_ESM.pdf]

Additional file 1. Main components of several balanced crystalloids.

Main components	NS	LRS	ARS[1]	ARS[2]	BRS	Plasma	ECF
Cl^-	154	111	98	115	109	100-106	117
Na^+	154	130	140	140	130	136-146	142
K^+	−	4	5	4	4	3.8-5.0	5
Mg^{2+}	−	−	1.5	1	1	1.3-2.1	1
Ca^{2+}	−	2	−	1.5	1.5	1.0-1.2	1.1-1.3
pH	5	6.5	7.4	5.3	7.3	7.35-7.45	7.35-7.45
Glucose (g/L)	−	−	−	10	−	3.9-6.0	−
Osmotic pressure (mOsm/L)	308	256-273	294	304	276	280-310	310
HCO_3^-<br>$Citrate^{3-}$	−	−	−	−	28<br>1.3	23-27	23-27
$Acetate^-$	−	−	27	25	−	−	−
$Lactate^-$	−	28	−	−	−	−	−

NS: Normal saline LRS: Sodium lactate Ringer's solution ECF: Extracellular fluid

ARS[1]: Sodium acetate Ringer's solution (Multiple Electrolytes Injection)

ARS[2]: Sodium acetate Ringer's solution (Sodium Potassium Magnesium Calcium and Glucose Injection)

BRS: Sodium bicarbonate Ringer's solution
